# Subretinal heterotopic respiratory epithelium: a case report and literature review

**DOI:** 10.1186/s13000-015-0416-8

**Published:** 2015-10-06

**Authors:** Ting Zhang, Hong Lin, Zhanyu Zhou, Nianting Tong, Ying Li, Yu Zhang, Liangyu Wang, Fuling Liu

**Affiliations:** Department of Ophthalmology, Qingdao Municipal Hospital, No.1 Jiaozhou Road, Shibei District, Qingdao, Shandong China; Department of Ophthalmology, the Affiliated Hospital of Qingdao University, No. 16 Jiangsu Road, Shinan District, Qingdao, Shandong China

## Abstract

A 51-year-old female underwent vitrectomy surgery to remove a group of spherical subretinal tumors beneath the detached retina. Hematoxylin and eosin staining and immunohistochemical findings showed that the characteristics of the tumor were consistent with a subretinal heterotopic respiratory epithelium. This is the first report of a respiratory epithelial heterotopia located in the subretinal space.

## Background

The choristoma, as a rare intraocular mass, is considered to be a congenital tumor that is not normally present in the area in situ. Although most ocular choristomas develop at the surface of the ocular, such as the conjunctiva, sclera, and orbit, the choristomas can also be found in intraocular structures, including the iris, ciliary body, retina, and optic nerve. Among them, the dermoid and epidermoid cysts are most common choristomatous cysts in the orbit.

Here, we present a case of a 51-year-old woman with a respiratory epithelial heterotopia located in the subretinal space for the first time. It is very likely to be a new type of intraocular choristoma.

## Case presentation

**Fig. 1:**
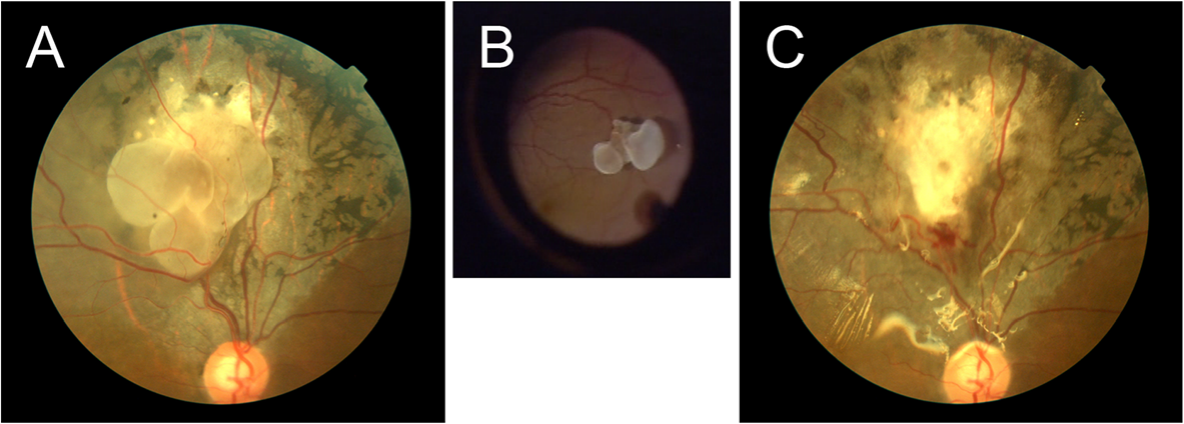
Fundus photographs before and after tumor removal. Photographs of the fundus are shown before (**a**) and after (**c**) tumor removal. The tumor was shown during the surgery (**b**)

**Fig. 2:**
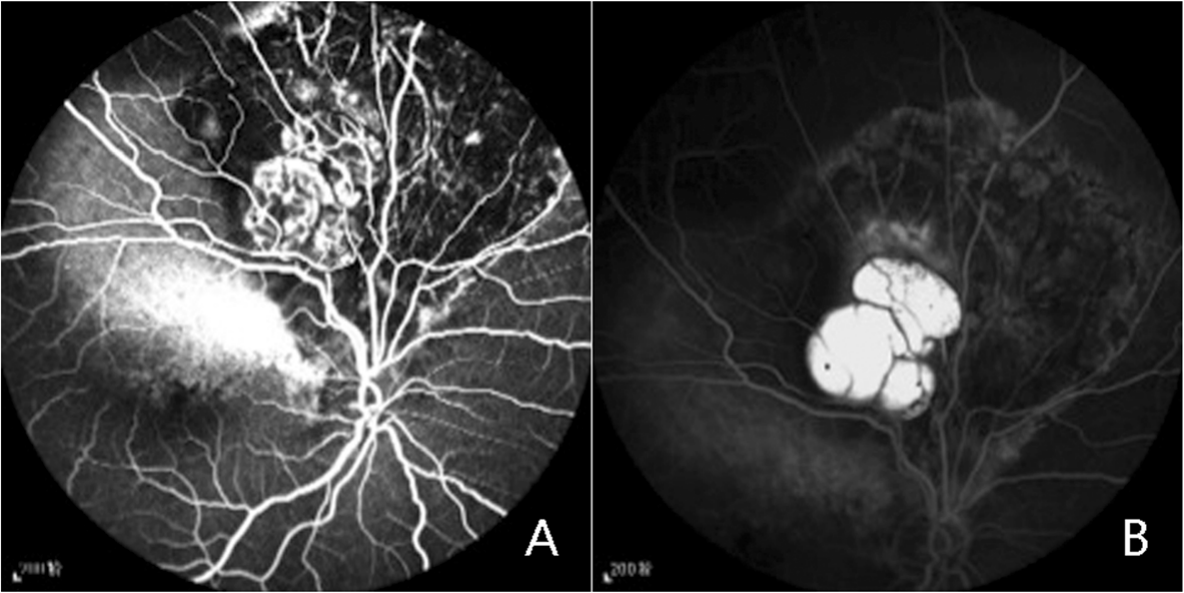
FFA photographs of the tumor. FFA results are shown in Fig. 2. The arteriovenous phase of FFA is shown in (**a**), and a large number of vascular networks inside the tumor, as well as the large choroidal vessels, can be seen around the tumor. In the later phase of FFA, the dye gradually leaked and the tumor accumulated fluorescein (**b**)

A 51-year-old postmenopausal female presented with the chief complaint of an inferior visual field defect in the right eye and decreased central vision lasting for one day without any other concomitant symptoms. The patient had no significant past medical history, and claimed to have no antecedent ocular trauma. Nothing abnormal was found upon general physical examination. Her best corrected visual acuity (BCVA) was 20/200, and the intraocular pressure was 18 mmHg. Nothing abnormal was found in the anterior segment, and the vitreous was clear, without cells or pigments. Biocular indirect ophthalmoscopy revealed focal retinal detachment involving the area from the macula to the supratemporal vascular arcades, with subretinal depigmentation and pigmentation. A group of spherical subretinal tumors was noted beneath the detached retina. The tumors were semi-translucent, pale, smooth-faced, lobulated, and stationary. Neither proliferous tissue nor retinal breaks were found in the area of retinal detachment (Fig. 1a). The subretinal fluid did not move with postural change. The results of fluorescein fundus angiography (FFA) were as follows: during the arteriovenous phase, large vascular networks inside the tumor could be seen, and due to a large area of choroid capillary atrophy, large choroidal vessels could be seen around the tumor (Fig. 2a). The fluorescein gradually accumulated in the tumor over time, but did not leak from the tumor into the subretinal fluid (Fig. 2b).

The initial diagnosis was exudative retinal detachment and a subretinal neoplasm in the right eye.

The retina reattached spontaneously seven days after hospitalization. To our surprise, the retina detached again and the subretinal fluid recurred in the same area three days after treatment with antibiotic eye drops. Visual acuity and intraocular pressure did not change. Due to the repeated recurrence of retinal detachment and the unknown nature of the subretinal neoplasm, the patient underwent vitrectomy surgery with informed consent. The subretinal neoplasm was removed, and the retina was reattached with a silicone oil intraocular tamponade during the operation. During the surgery, the subretinal neoplasm was found to be spherical, lobulated and smooth-faced, and to be connected to the white fibrotic tissue under the tumor by a slender peduncle without any synechia to the retina (Fig. 1b). The tumor was soft, deformable and elastic. The dendritic vascular network could be clearly detected inside the tumor when transilluminated with an optical fiber. The tumor was completely removed from the eye after cutting the peduncle, and was then fixed in formaldehyde. The fundus is shown after removal in Fig. 1c.

**Fig. 3:**
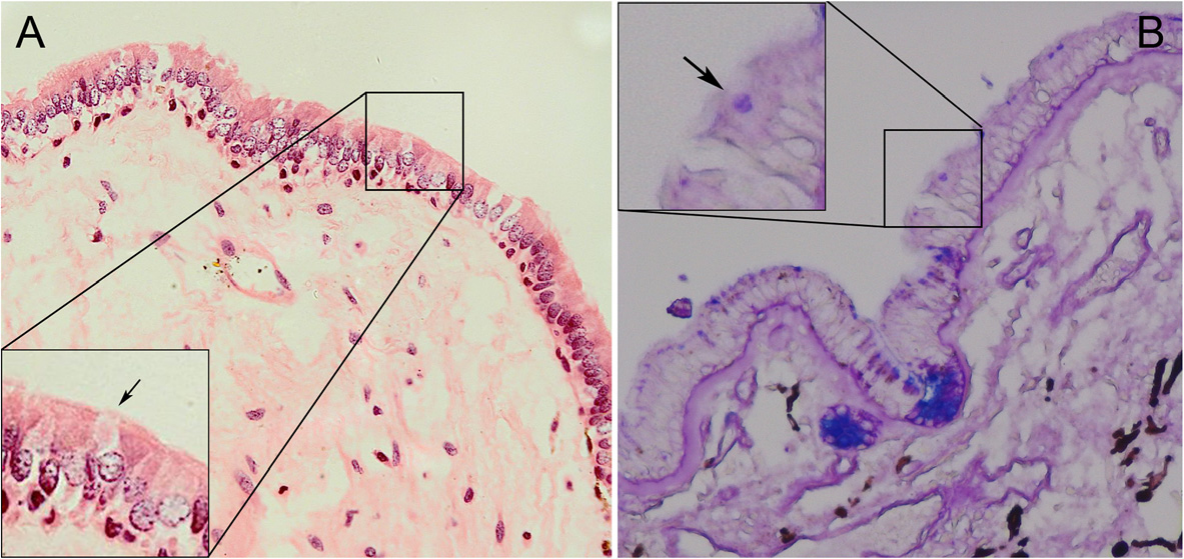
Specimen pathological examination. The solid tumor, which was covered with pseudostratified ciliated columnar epithelium and contained scattered goblet cells, is shown in (**a**) by HE staining (representative goblet cell was shown by arrow) and in (**b**) by Alcian blue stain (representative goblet cell was shown by arrow)

**Fig. 4:**
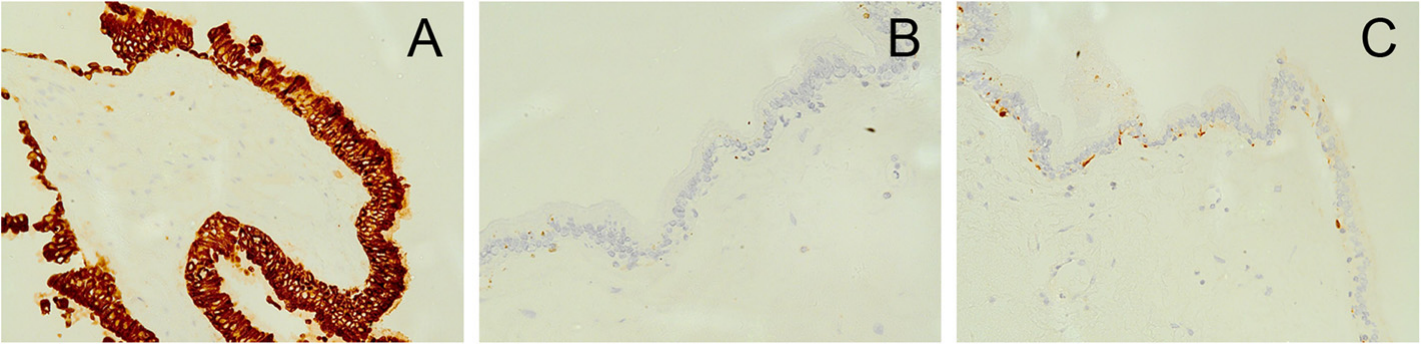
IHC staining for CK, GFAP and S100. The strong positive staining of CK is shown in (**a**). While negative staining images of GFAP and S100 are shown in (**b**) and (**c**), respectively

The excised mass was fixed in 10 % formalin and embedded in paraffin. Several 6-μm sections were cut from each paraffin block, two sections were stained with hematoxylin and eosin (H&E) and periodic acid Schiff-Alcian blue, and the others were stained with various antibodies (immunohistochemistry (IHC)). Immunohistochemical staining was performed using the streptavidin-peroxidase system (Ultrasensitive; Mai Xin Inc., Fuzhou, China) according to the manufacturer’s instructions. Commercially available, pre-diluted monoclonal antibodies against the following antigens were used: cytokeratin (CK), nerve tissue protein S100, glial fibrillary acidic protein (GFAP) (all Thermo Fisher Scientific Inc., Fremont, CA, USA). H&E staining revealed that the tumor was solid and covered with pseudostratified ciliated columnar epithelium containing scattered goblet cells, which were prominently shown by Alcian blue stain (representative goblet cell was shown in Fig. 3a and b by arrow). There was loose connective tissue just beneath the epithelia, which contained blood vessels, fibroblasts, fibrocytes, lymphocytes, plasmocytes, and macrophages. IHC was positive for CK (Fig. 4a), but negative for S100 (Fig. 4b) and GFAP (Fig. 4c).

### Diagnosis

Histologically, the tumor was composed of pseudostratified ciliated columnar epithelium and scattered goblet cells. IHC was positive for CK and negative for S100 and GFAP. Therefore, the tumor was diagnosed as a subretinal heterotopic respiratory epithelium.

### Differential diagnosis

To avoid overtreatment, including chemotherapy and radiotherapy, differentiating this tumor from other malignant tumors, including amelanotic malignant melanomas and metastatic carcinomas, was important.

Amelanotic malignant melanomas are rare, lack pigment, and have round edges like the tumor found in this patient. In addition, the FFA results found in patients with malignant melanomas are typically similar to those in this case. However, intraocular malignant melanomas may extend into adjacent intraocular tissues or outside the eye through the scleral canals, or by intravascular invasion. Furthermore, malignant melanomas are composed of spindle-shaped cells, with or without prominent nucleoli, and large epithelioid tumor cells. There was no evidence of adjacent tissue invasion nor typical spindle-shaped cells in this case.

Due to the rich blood supply of the choroid, it is an important site for blood-borne metastases. In females, carcinoma of the breast is the most common source, while in males, lung, liver, genitourinary, and gastrointestinal malignancies are the usual primary sites. Metastatic carcinomas can have a similar appearance as the tumor in this case, including being pale, smooth-faced and lobulated, and are also stationary. Meanwhile, carcinomas often have serous retinal detachment, which is again, similar to the tumor described in this case. In patients suffering from metastatic carcinomas, metastasis to the choroid usually becomes apparent after diagnosis of the primary malignancy. However, the appearance of a choroidal metastasis may occasionally precede the diagnosis of the primary neoplasm, but upon histological examination, malignant cells can be found in the tissue sections. In this patient, nothing abnormal was found through a detailed general physical checkup, and no malignant cells were found in the tumor.

Also, it is necessary to differentiate this tumor from some benign tumors, including choroid hemangiomas and choroid osteomas.

Choroid hemangiomas and choroid osteomas can appear similar to this tumor in fundus examinations. Especially in choroid hemangiomas, which is more dome-shaped and often has a serous retinal detachment, it is difficult to identify only in fundus examination. Fluorescein Angiography is an important examination to identify the choroidal hemangioma. Typically, during the early phase, there are irregular areas of hyper-fluorescence in choroid hemangiomas. This hyper-fluorescence reflects the filling of vascular spaces within the tumor. This separates the choroidal hemangiomas from other solid, vascular tumors. A persistent hyper-fluorescence in the late phase can be caused by an accumulation of fluorescein in the vascular spaces of the hemangioma, or by pooling of the dye in subretinal fluid.

Choroidal osteomas are benign tumors of the choroid that are composed of mature bone. These tumors are typically found in healthy young females in the second or third decades of life, are relatively flat, and have pseudopod-like processes on the edges of the lesion. The diagnosis of choroidal osteoma was ruled out in this case since no osteocytes were found in the tissue section.

## Discussion

This case is the first report of an intraocular respiratory epithelial heterotopia. It was also the first report of forming the solid tumors appearance secondary to the respiratory epithelial heterotopia. However, the reason for the formation of a tumor with a simple structure covered by epithelial cells remains unclear.

The specimen from this patient showed typical pseudostratified ciliated columnar epithelium containing goblet cells, which was confirmed by examining the histologic features of the respiratory epithelium. IHC also showed that the tumor originated from the covering epithelium rather than the neural epithelium. In addition, the loose connective tissues, without any involvement from the mucosal gland or smooth muscle structure under the epithelium, suggest that it was a simple respiratory epithelium ectopic instead of a mucosa ectopic.

At present, the majority of respiratory epithelium ectopic diseases in the eye have been reported in the orbit and the outer surface of the cornea limbus [1–6]. In these cases the lesions were cysts, had ectopic pseudostratified ciliated columnar epithelium in the inner surface, and the cavities were filled with mucous. However, the respiratory epithelial heterotopia in this case was located in the subretinal space with a lobulated, solid appearance, and was connected to the underlying fibrous tissue by a slender peduncle through which blood was supplied.

A large area of hyperpigmentation and depigmentation around the tumor, the atrophy of the capillary and middle blood vessels of the choroid, and the fibroplasia showed that the lesion had persisted for a long period of time. Although it was uncertain whether the retinal detachment was rhegmatogenous or exudative, the repeating occurrence of retinal detachment and the absence of retinal breaks during vitrectomy suggested an exudative retinal detachment. FFA showed the accumulation of fluorescein in the tumor without any leakage into the subretinal fluid. However, FFA was performed when the subretinal fluid was spontaneously absorbed, and retinal detachment was aggravated two days later. Therefore, whether the subretinal fluid came from tumor leakage or from goblet cell secretion is still unclear.

The possible mechanisms of heterotopic respiratory epithelium tumor formation are still unclear. The reported heterotopic locations of respiratory epithelium include the lingua [7], mandible [8], eyes [6, 9], rectum [10] and thyroid [11]. All were cystic lesions except the rectum. There have been 15 cases of ophthalmic heterotopic respiratory epithelium reported so far; 14 were in the orbit, and one was in the limbus [6]; seven cases were associated with trauma or surgery, while eight cases were considered to be choristomas.

A choristoma is defined as a congenital tumor of tissues that are not normally present in the involved area [12]. It is the most common ocular surface and orbital tumor-like lesion in children. The representative species include ophthalmic epidermoid tumor, orbital endothelial cysts and epidermoid cysts. Choroid osteoma is also a common ophthalmic choristoma. Locations of reported ophthalmic choristoma include the orbit [5], ocular surface (conjunctiva [13], cornea [13], limbus [6], subconjunctiva and sclera [12]), inner eye (iris and ciliary body [14], choroid and retina [12]), ocular adnexa (extraocular muscles [15], acrimal gland and eyelid [16]), and optic nerve [9]. The tissues include the skin, conjunctival tissue, respiratory epithelial tissue, lacrimal tissue, cerebral tissue, neuromuscular tissue, osteal and cartilaginous tissue, adipose and smooth muscle tissue, and tissue deriving from the lens.

Choristomas may be derived from heterotopic pluripotent cells or normal tissue, which is crowded out of its normal locations during development, and is subsequently found in abnormal areas of the embryo. Pseudostratified ciliated columnar epithelium containing goblet cells is mainly distributed in the respiratory tract, including the mucosal surface of the nasal cavity, paranasal sinus, larynx, trachea, and bronchus. The mucosal surface of the paranasal sinus is in close proximity to the eye, and is considered the most likely source of ocular heterotopic respiratory epithelium. In addition, heterotopic respiratory epithelium has also been reported in some branchial cysts. The first 4 to 8 weeks of embryonic development are important for the development of the eyes, nose and face. The heterotopic epithelium of the original nasal mucosa may remain around the developing eyeball and eventually form a heterotopic respiratory epithelial cyst or choristoma. The sclera appears during the eighth week of embryonic development, and is well-developed during the fifth month. The orbital bone forms during the eighth week of embryonic development, but prior to this stage, the epithelium of the original nasal cavity is close to the orbital and optic cup, without the obstruction of the orbital bone or compact sclera. Hence, this tissue likely grows near the outside surface of the optic cup.

Half of the reported orbital heterotopic respiratory epithelium cysts were related to orbital trauma or reconstructive surgery following orbital fracture, which may be implanted cysts. However, this patient had no antecedent trauma or facial surgery, and orbital computed tomography (CT) showed a complete orbital bone and paranasal sinus. Therefore, an implanted cyst can be ruled out.

## Conclusions

In summary, we report a rare case of subretinal heterotopic respiratory epithelium, which is confirmed by H&E staining, Alcian Blue staining, and IHC staining for CK, GFAP and S100. This case is the first report of an intraocular respiratory epithelial heterotopia, and the most likely reason for the formation of this tumor is an intraocular choristoma.

## Consent

Written informed consent was obtained from the patient for publication of this case report and any accompanying images. A copy of the written consent is available for review by the Editor-in-Chief of this journal.

## References

[1] Jakobiec FA, Roh M, Stagner AM, Yoon MK. Choristomatous Respiratory Cyst Restricted to the Upper Eyelid. Ophthalmic plastic and reconstructive surgery 2015. 10.1097/IOP.0000000000000373 25675165

[2] Thanos A, Jakobiec FA, Mendoza PR, Hatton MP (2014). Ectopic (choristomatous) orbital respiratory cyst: histopathology and immunohistochemistry. Surv Ophthalmol.

[3] Tay E, Yee AC, Luthert PJ, Rose GE (2014). Congenital respiratory epithelial cysts of the orbit: a rare cause of major orbital impairment. Ophthal Plast Reconstr Surg.

[4] Mee JJ, McNab AA, McKelvie P (2002). Respiratory epithelial orbital cysts. Clin Experiment Ophthalmol.

[5] Morris WR, Fleming JC (2001). Respiratory choristomatous cysts in the temporal orbit. Ophthal Plast Reconstr Surg.

[6] Young TL, Buchi ER, Kaufman LM, Sugar J, Tso MO (1990). Respiratory epithelium in a cystic choristoma of the limbus. Arch Ophthalmol.

[7] Mandell DL, Ranganathan S, Bluestone CD (2002). Neonatal lingual choristoma with respiratory and gastric epithelium. Arch Otolaryngol Head Neck Surg.

[8] Noorchashm N, Huff DS, Bartlett S (2004). A mixed heterotopic gastrointestinal and respiratory cyst of the oral cavity with an intraosseous component. Plast Reconstr Surg.

[9] Giannini C, Reynolds C, Leavitt JA, Schultz GA, Garrity JA, Ebersold MJ (2002). Choristoma of the optic nerve: case report. Neurosurgery.

[10] Kawahara K, Mishima H, Nakamura S (2007). Heterotopic respiratory mucosa in the rectum: a first case report. Virchows Arch.

[11] Park JY, Kim GY, Suh YL (2004). Intrathyroidal branchial cleft-like cyst with heterotopic salivary gland-type tissue. Pediatric Dev Pathol.

[12] Kim BH, Henderson BA (2005). Intraocular choristoma. Semin Ophthalmol.

[13] Hayasaka S, Sekimoto M, Setogawa T (1989). Epibulbar complex choristoma involving the bulbar conjunctiva and cornea. J Pediatr Ophthalmol Strabismus.

[14] Shields JA, Hogan RN, Shields CL, Eagle RC, Weakley DR (2000). Intraocular lacrimal gland choristoma involving iris and ciliary body. Am J Ophthalmol.

[15] Suh MH, Kim JH, Kim SJ, Yu YS (2008). Osseous choristoma of an extraocular muscle. J AAPOS.

[16] Gordon AJ, Patrinely JR, Knupp JA, Font RL (1991). Complex choristoma of the eyelid containing ectopic cilia and lacrimal gland. Ophthalmology.

